# What makes a RAG regeneration associated?

**DOI:** 10.3389/fnmol.2015.00043

**Published:** 2015-08-07

**Authors:** Thong C. Ma, Dianna E. Willis

**Affiliations:** ^1^Department of Neurology, Columbia UniversityNew York, NY, USA; ^2^Brain Mind Research Institute, Weill Cornell Medical CollegeNew York, NY, USA; ^3^Burke-Cornell Medical Research InstituteWhite Plains, NY, USA

**Keywords:** regeneration, regeneration associated genes, injury conditioning, cyclic AMP, transcription factors

## Abstract

Regenerative failure remains a significant barrier for functional recovery after central nervous system (CNS) injury. As such, understanding the physiological processes that regulate axon regeneration is a central focus of regenerative medicine. Studying the gene transcription responses to axon injury of regeneration competent neurons, such as those of the peripheral nervous system (PNS), has provided insight into the genes associated with regeneration. Though several individual “regeneration-associated genes” (RAGs) have been identified from these studies, the response to injury likely regulates the expression of functionally coordinated and complementary gene groups. For instance, successful regeneration would require the induction of genes that drive the intrinsic growth capacity of neurons, while simultaneously downregulating the genes that convey environmental inhibitory cues. Thus, this view emphasizes the transcriptional regulation of gene “programs” that contribute to the overall goal of axonal regeneration. Here, we review the known RAGs, focusing on how their transcriptional regulation can reveal the underlying gene programs that drive a regenerative phenotype. Finally, we will discuss paradigms under which we can determine whether these genes are injury-associated, or indeed necessary for regeneration.

It likely comes as no surprise the striking dichotomy that exists between peripheral nervous system (PNS) and central nervous system (CNS) neurons following injury. While PNS neurons show robust regenerative capacity, CNS neurons exhibit negligible capacity. This difference has been known and intensively studied since the time of Ramón y Cajal, and in those years many reasons have been postulated for this fundamental difference (Ramón y Cajal, 1914). What then have we learned from nearly 100 years and hundreds of studies trying to unravel this mystery? Deservingly, the gene transcription response to axon injury has drawn considerable interest; however, its increasingly appreciated complexity poses a formidable challenge from a therapeutic perspective. Given this potential hurdle, ongoing work has given hope that if the appropriate manipulations of neuronal physiology are enacted, both the central and PNS could be efficiently repaired.

## What Evidence Pointed to the Importance of Regeneration Associated Genes (RAGs)?

Regeneration of damaged axons is dependent on the neuron-intrinsic transcription of regeneration-associated genes (RAGs). Axotomy of PNS neurons induces broad and coordinated gene transcription, a response that is lacking following CNS injury (Schreyer and Skene, 1993; Mason et al., 2002; Starkey et al., 2009; Ylera et al., 2009; Geeven et al., 2011). These differences in the ability to induce RAG expression, along with extracellular environmental factors, underlie the disparate ability of PNS and CNS axons to regenerate (Filbin, 2003).

Early observations that peripheral axon injury induced a “cell body” response that included increased neuronal mRNA and protein synthesis indicated an active process by which peripheral neurons *prepared* to regenerate axons (Lieberman, 1971; Grafstein, 1975). Along with findings that specific axonal proteins were upregulated following injury (i.e., GAP43), the idea that the expression of growth-related proteins promoted the regeneration of axons began to take hold (Skene and Willard, 1981; Skene, 1989; Tetzlaff et al., 1991). As a result of these early observations, the hypothesis formed that injury-induced gene transcription was required for axon regeneration, and importantly, raised the possibility that the expression of RAGs may confer regenerative capacity to CNS neurons.

This brought to question whether the primary driver of regenerative failure in the CNS was due to the inhibitory environment or the failure to appropriately upregulate RAGs. If the latter, it suggested that a reasonable course of action to confer regeneration capacity to the CNS was to identify and manipulate the RAGs responsible for the PNS response.

## What Constitutes a RAG?

With the early evidence suggesting that the regenerative transcriptional response could be used to improve regeneration, both under permissive and non-permissive conditions, considerable effort has been directed at identifying the genes that are upregulated following injury and designing methods to modulate their expression to enhance regeneration in CNS neurons.

Several seminal observations supported the existence of neuron-intrinsic factors capable of promoting CNS regeneration. Though typically incapable of spontaneous regeneration, CNS neurons will regenerate damaged axons when provided a permissive environment. Indeed, some damaged spinal cord axons grow into transplanted peripheral nerve segments in the rat spinal cord, indicating that these CNS neurons retained the intrinsic capacity to regenerate given a permissive (or growth-stimulating) environment (David and Aguayo, 1981). Interestingly, though not all types of CNS neurons exhibit this behavior, those that could regenerate upregulate RAG expression in the presence of the graft (Anderson et al., 1998; Mason et al., 2002; Murray et al., 2011).

Manipulations that increase RAG expression in CNS can also promote regeneration of “resistant” axons into these nerve grafts. For instance, treatment with BDNF of rubrospinal neurons induces RAG expression and growth into peripheral nerve grafts, while upregulating cyclic adenosine monophosphate (cAMP) levels can increase RAG expression and allow modest CNS axon regeneration in *in vivo* CNS injury models (Kobayashi et al., 1997; Ye and Houle, 1997; Neumann et al., 2002; Qiu et al., 2002; Li et al., 2003; Storer et al., 2003; Jin et al., 2009). Indeed, cAMP is one of the few manipulations that has repeatedly been shown to drive axon regeneration in a variety of CNS injury models performed by numerous research groups.

Dorsal root ganglia (DRG) neurons have provided an important *in vivo* platform to test whether RAG induction allows regeneration of CNS axons. These sensory neurons possess pseudounipolar axons that extend in the periphery and into the spinal cord; a subset of these axons ascend the dorsal column of the spinal cord (Bradbury et al., 2000). Peripheral nerve injury (transection or crush) induces the expression of RAGs, whereas injury to the central projecting branch does not (Schreyer and Skene, 1993; Smith and Skene, 1997; Mason et al., 2002; Hanz et al., 2003; Seijffers et al., 2006; Ylera et al., 2009; Geeven et al., 2011). Intriguingly, a peripheral *conditioning* lesion enhances regeneration of proximally reinjured peripheral axons, and allows regeneration of a subsequently injured central branch (McQuarrie and Grafstein, 1973; McQuarrie et al., 1977; Oblinger and Lasek, 1984; Neumann and Woolf, 1999). These observations have led to substantial research efforts aimed at understanding this mechanism. This conditioning lesion effect was shown to be transcription dependent, indicating that the physiological induction of RAG transcription confers regenerative capacity to an otherwise regeneration-deficient axon (Smith and Skene, 1997). Together, these observations indicate that the expression of genes induced by peripheral axon injury are necessary for spontaneous regeneration and the forced expression of these (by peripheral injury) can be sufficient to drive regeneration of CNS axons.

**Figure 1:**
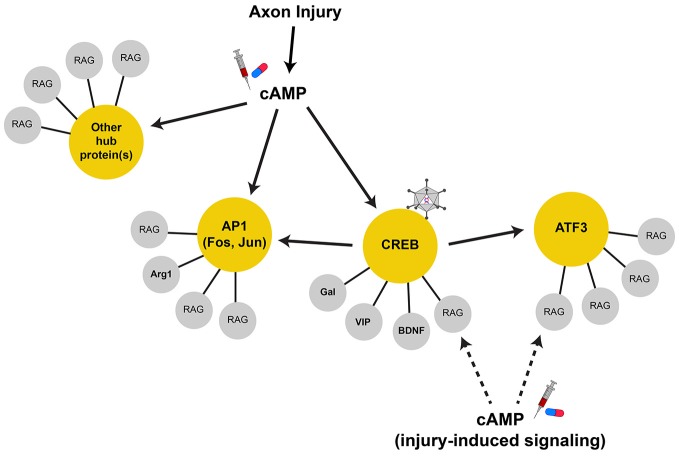
**Regeneration-associated gene networks**. Axonal injury activates many signal transduction pathways that can lead to gene transcription. The upregulation of cAMP levels after injury is important for RAG expression, serving to activate CREB, AP1, and possibly other transcription factors in parallel. These transcription factors can serve as hub proteins (in yellow circles) to control the transcription of terminal RAGs (in gray circles) that may serve related physiological functions. Some hub proteins, such as CREB, drive the transcription of other hub proteins. In this case, AP1 subunits and ATF3 are direct CREB target genes. As such, CREB is a highly connected node of the RAG transcription network and serves to coordinate the transcription of many terminal RAGs through their proximal hub proteins. These highly connected nodes are attractive therapeutic targets that can recapitulate more of the RAG response and can be targeted by viral-mediated gene delivery (i.e., constitutive-active CREB, virus cartoon). Additionally, injury-induced signals may also work locally and interact with the protein products of the transcribed RAGs to augment axon growth. Thus, strategies that increase/induce RAG expression along with activation of injury signals (i.e., cAMP, syringe and pill cartoon) may show synergy in promoting axon regeneration.

Though many candidate RAGs have been identified, genome-wide profiling studies have provided a comprehensive view of the transcriptional changes that result from peripheral axon injury (Zigmond et al., 1998; Costigan et al., 2002; Xiao et al., 2002; Schmitt et al., 2003; Tanabe et al., 2003; Küry et al., 2004; Di Giovanni et al., 2005; Bosse et al., 2006; Stam et al., 2007; Szpara et al., 2007; Moore et al., 2009; Zou et al., 2009; Michaelevski et al., 2010; Geeven et al., 2011; Ma et al., 2011; Blesch et al., 2012). From these, it is apparent that the expression levels of thousands of genes are changed by injury. Elucidating the identity and function of intrinsic contributors to regeneration has been aided by numerous studies performed in lower vertebrates and invertebrates, which offer advantages of forward genetic screens and *in vivo* imaging, and complement those studies performed in mammals. They offer insights into how injury-induced gene expression both recapitulates and differs from pathways involved during development. For example, screening 654 conserved genes in an axotomy model of mechanosensory neurons in *C. elegans* identified clusters of genes that promote or repress axon growth. Many of these are components of pathways critical for neuronal plasticity of both development and regeneration; however, some clusters are only required for regeneration (Chen et al., 2011). This highlights how the repair process utilizes plasticity mechanisms important for neuronal development, but also has members unique to the repair process. As with development and many other biological processes, these genes function in regulatory networks and are interconnected by their interactions with other genes (Figure 1). Genes in a network can be “connected” in various ways, including by experimental evidence for physical interaction of the gene products (proteins), common gene function, or predicted regulation by signaling pathways or transcription factors. Because the gene network induced by injury is large and may contain redundant and/or complimentary genes, manipulating singe (or small subsets) of terminal genes is unlikely to recapitulate the effect of the entire regenerative “program.” Equally unlikely is our ability to experimentally recapitulate the entirety of the injury response.

Genes that have many connections act as “hubs” that coordinate the expression or activity of the connected genes (Batada et al., 2006; Van Kesteren et al., 2011; Song and Singh, 2013). Transcription factors serve as hubs in gene regulatory networks, and as such, are attractive targets for the manipulation of many genes that may sub serve common functions (Figure 1). Transcription-associated networks can be built by connecting genes based on the presence of promoter transcription factor binding sites *in silico* (Michaelevski et al., 2010; Geeven et al., 2011). Along with profiling of the injury-induced phosphoproteome to determine the associated signaling pathways activated, one study has distilled the RAG network to ~40 transcription factors downstream of multiple parallel signaling pathways (Michaelevski et al., 2010). Other independently identified transcription factors include ATF3, c-Jun, C/EBPβ, CREB, KLF family members, p53, SMAD1, SOX11, and STAT3 among others (Jenkins and Hunt, 1991; Herdegen et al., 1997; Schwaiger et al., 2000; Tsujino et al., 2000; Schweizer et al., 2002; Gao et al., 2004; Nadeau et al., 2005; Jankowski et al., 2006; Okuyama et al., 2007; Moore et al., 2009; Zou et al., 2009). It should be noted that the expression levels of some transcription factors might not be upregulated by injury (i.e., CREB and p53); however, their activity is induced and contribute to the overall RAG response (Gao et al., 2004; Tedeschi et al., 2009). While this number of pathways/transcription factors remains daunting, it brings to question whether the entire injury response is necessary to facilitate regeneration, and whether all injury-induced genes are necessarily RAGs (Table 1).

**Table 1:** **Regeneration-associated transcription: RAGs and genetic manipulations that alter axon regeneration after injury *in vivo***.

Gene (s)	Regeneration phenotype after injury	Reference
**Terminal RAGs**
α7 integrin	Knockout delayed facial nerve regeneration	Werner et al. (2000)
BDNF	Conditional knockout decreased sciatic nerve axon growth into peripheral nerve graft; adenoviral overexpression in sensorimotor cortex neurons increased CST axon sprouting/midline crossing	Zhou and Shine (2003) and English et al. (2013)
β2-microglobin	Knockout decreased sciatic nerve motor axon regeneration	Oliveira et al. (2004)
Cdc42	Delivery of constitutive-active protein by hydrogel increased CST tract axon growth through spinal cord lesion	Jain et al. (2011)
CGRP	Local siRNA against CGRP at site of axon injury reduced regeneration of sciatic nerve	Toth et al. (2009)
CRMP2	Adenoviral overexpression increased hypoglossal motor axon regeneration	Suzuki et al. (2003)
Galanin	Knockout decreased sciatic nerve regeneration	Holmes et al. (2000)
Galectin-1	Knockout delayed functional recovery of whisker movement after facial nerve crush; allograft treated with recombinant-oxidized galecitin-1 increased axon regeneration into sciatic nerve	Fukaya et al. (2003) and McGraw et al. (2004)
GAP43 + CAP23	Double transgenic overexpression increased central sensory axon regeneration into a peripheral nerve graft in the spinal cord	Bomze et al. (2001)
GDNF	Transplanted fibroblasts expressing GDNF at spinal cord transection sites increased spinal cord axon regeneration	Blesch and Tuszynski (2003)
Hsp27	Transgenic overexpression increased sciatic nerve motor and sensory axon growth rate and functional recovery	Ma et al. (2011)
IL6	Knockout delayed sciatic nerve regeneration; intrathecal injection of IL6 increased dorsal column sensory axon regeneration	Zhong et al. (1999) and Cao et al. (2006)
p21^cip1/WAF1^	Knockout delayed sciatic nerve motor axon regeneration and functional recovery	Tomita et al. (2006)
PACAP	Knockout decreased facial nerve regeneration; PACAP delivery by hydrogel increased regenerating axons in contusion model of SCI	Armstrong et al. (2008) and Tsuchida et al. (2014)
Rac1	Delivery of constitutive-active protein by hydrogel increased CST axon growth through spinal cord lesion	Jain et al. (2011)
**Regeneration-associated transcription factors**
ATF3	Overexpression increased sciatic nerve regeneration	Seijffers et al. (2007)
C/EBPdelta	Knockout delayed sciatic nerve regeneration	Lopez De Heredia and Magoulas (2013)
c-Jun	Knockout reduced facial nerve reinnervation and delayed functional recovery	Raivich et al. (2004)
CREB	Adenovirus overexpression of constitutive-active CREB in DRG neurons increased dorsal column sensory axon regeneration	Gao et al. (2004)
KLF7	AAV overexpression of constitutive-active KLF7 of sensorimotor cortex neurons increased CST tract axon regeneration	Blackmore et al. (2012)
p53	Knockout decreased number of regenerating facial nerve axons	Tedeschi et al. (2009)
SMAD1	Increasing SMAD1 activity with BMP4 in DRG neurons increased dorsal column axon regeneration	Parikh et al. (2011)
SnoN	AAV overexpression of degradation-resistant SnoN in DRG neurons increased dorsal column axon regeneration	Do et al. (2013)
SOX11	Knockdown with membrane-permeable siRNA decreased sciatic nerve regeneration; HSV overexpression accelerated saphenous nerve regeneration	Jankowski et al. (2009) and Jing et al. (2012)
STAT3	Knockout in DRG neurons decreased the initiation of regenerating peripheral axons while AAV overexpression increases terminal sprouting of dorsal column axons; AAV overexpression in motor cortex increased CST axon sprouting	Bareyre et al. (2011) and Lang et al. (2013)

*AAV, adeno-associated virus; CST, cortical spinal tract; HSV, herpes simplex virus; SCI, spinal cord injury, siRNA, small-interfering RNA*.

Individually identified (not via microarray) non-transcription factor “terminal” RAGs span many functional categories, including genes that encode adhesion/guidance molecules (i.e., integrin subunits, CD44; Kloss et al., 1999; Jones et al., 2000), neuropeptides (i.e., VIP, Gal, CGRP, NPY, PACAP, etc.; Mohney et al., 1994; Holmes et al., 2000; Suarez et al., 2006; Sachs et al., 2007; Armstrong et al., 2008; Toth et al., 2009), structural and cytoskeletal-associated proteins (i.e., GAP43, CAP23, SCG10, CRMP2, Sprr1a; Skene and Willard, 1981; Bomze et al., 2001; Bonilla et al., 2002; Iwata et al., 2002; Mason et al., 2002; Suzuki et al., 2003), and metabolic enzymes (i.e., arginase 1; Cai et al., 2002). The contribution of specific RAGs to axon regeneration/growth (either in the PNS or CNS neurons) has been assessed both *in vitro* and *in vivo* by evaluating their sufficiency or necessity for the regenerative response. Necessity is addressed by either pharmacological inhibition or genetic attenuation/deletion of the candidate RAG. These studies often show that knockdown of individual downstream RAGs results in small decreases, or delays, in peripheral regeneration. By contrast, deletion of possible hub proteins (transcription factors or kinases) leads to more dramatic effects *in vivo*. For example, genetic knockout of c-Jun, the first identified transcription factor RAG, reduced re-innervation of peripheral targets by 4-fold after facial nerve axotomy and was associated with decreased induction of its downstream target RAGs α7 integrin, CD44, and galanin (Herdegen et al., 1991; Jenkins and Hunt, 1991; Raivich et al., 2004). By contrast, deletion of α7 integrin or galanin only delayed target re-innervation and functional recovery (Holmes et al., 2000; Werner et al., 2000). This dichotomy also holds true for the above-mentioned terminal RAGs and hub transcription factors. Taken together, these studies suggest that the modulation of any single downstream RAG is unlikely to have robust effects on overall regeneration.

Similarly, sufficiency experiments have yielded mixed effects as the overexpression of terminal RAGs or transcription factors can recapitulate some aspects of peripheral nerve injury, but none capture the phenotypic entirety of the regenerative “program.” For instance, though there was much interest in GAP43 as a RAG, its overexpression failed to promote regeneration in many CNS injury models, though it induced significant sprouting (Buffo et al., 1997; Harding et al., 1999; Mason et al., 2000). Instead, the co-expression of both GAP43 and CAP23, two related growth cone proteins, was required to drive regeneration into a peripheral nerve graft in the spinal cord (Bomze et al., 2001). While the overexpression of some hub transcription factors facilitated *in vivo* regeneration, these effects were also mixed. One example was ATF3, whose overexpression afforded only increased PNS (but not CNS) axon regeneration *in vivo*. Further, ATF3 overexpression did not allow axon growth on inhibitory substrates *in vitro*, suggesting that ATF3 increases the intrinsic growth of axons, but does not alter its response to the inhibitory environment of the CNS (Seijffers et al., 2007). By contrast, STAT3 overexpression seems to allow the initiation of CNS axon growth, but does not sustain its elongation (Bareyre et al., 2011). Taken together, the disparate effects of targeting transcription factors also suggests that each may play specific roles in the regeneration process, and that several hubs may need to be manipulated for a sufficient response (Figure 1). Table 1 lists manipulations of terminal RAGs and regeneration-associated transcription factors that have been shown to impact axon growth *in vivo*.

Interestingly, some injury-induced genes may actually oppose axon growth. For instance, though both SOCS3, which suppresses cytokine signaling, and NFIL3, a transcription factor, are strongly induced by peripheral injury, their deletion or attenuation promotes axon growth (Miao et al., 2006; MacGillavry et al., 2009; Smith et al., 2009). Overexpression of SOCS3 further decreased axon growth, supporting that not all injury-induced genes are necessarily RAGs, and that attenuating negative regulators of axon growth may be beneficial (Miao et al., 2006). This further underscores the notion that not every component of the injury response is contributing to regeneration.

## Sufficiency for Therapeutics?

As the changes in gene transcription following axon injury are broad, how do we assess whether they are truly regeneration-associated? As discussed, though many necessity studies show the contribution of candidate injury-induced RAGs to the regenerative response, the sufficiency of their expression in driving regeneration is typically modest. Therapeutic approaches must leverage the sufficiency of genes and transcriptional pathways, organized into larger hubs or programs, to drive axon growth and regeneration. For this purpose, genes and pathways that may not be *normally* recruited by injury could also be considered.

Given that many of the pro-regenerative pathways identified are involved in the neuronal plasticity during development, one major effort has been to reconstitute certain aspects of a “younger” developmental state in which CNS neurons are axon growth competent. These studies sought specific genes, transcription factors, or signaling pathway components that were changed between developmental epochs where CNS neurons lose their regenerative capacity. For example, the activation of Raf-MEK-ERK pathway that is a dominant driver of developmental axon growth downstream of growth factor signaling can also drive robust CNS axon growth in adult neurons (Hollis et al., 2009; O’Donovan et al., 2014). Other developmentally-regulated targets that, when modulated, increase CNS axon growth include transcription factors (KLF family, SnoN; Moore et al., 2009; Blackmore et al., 2012; Do et al., 2013), transcriptional/epigenetic regulators (Set-B and P300; Tedeschi et al., 2009; Trakhtenberg et al., 2014), and others (let-7 microRNA; Zou et al., 2013). It should be noted that some negative regulators of axon regeneration, such as KLF4, are upregulated during development in CNS neurons; attenuation of these genes, as with injury-induced inhibitors of axon growth, may be significant adjuncts to driving growth-associated targets (Moore et al., 2009).

Another fruitful strategy has been to increase the “metabolic growth state” of CNS neurons, focusing on upregulating anabolic processes such as protein translation through mTOR activation [i.e., Phosphatase and tensin homolog (PTEN) deletion or Rheb activation] or transcriptional regulation of anabolic processes (i.e., c-myc overexpression), all of which increase CNS axon growth after injury (Park et al., 2008; Liu et al., 2010; Kim et al., 2011; Belin et al., 2015). While these processes may decline with development in CNS neurons, augmenting these pathways also counteracts some injury-induced deficits such as mTOR and c-myc activity reduction, and is thought to provide the metabolic and energetic substrates required for axon growth (Park et al., 2008; Belin et al., 2015). These studies have also led to combinatorial approaches that pair increased metabolic state (PTEN deletion) with other manipulations that increase axon growth. Indeed, the co-deletion of PTEN and SOCS3, co-deletion of PTEN and SOCS3 plus c-myc overexpression, and deletion of PTEN plus BRaf activation have yielded highly robust long distance axon growth of injured optic nerves, with evidence for synergistic interaction of each manipulation (Sun et al., 2011; O’Donovan et al., 2014; Belin et al., 2015).

Though not necessarily induced by peripheral axon injury, the action of epigenetic modifiers has gained significant interest in facilitating RAG transcription. This is especially true given the difficulty in directly upregulating gene expression *in vivo*, which likely limits the therapeutic potential of these types of approaches. Epigenetic modification of DNA or DNA/protein complexes of chromatin can dictate the transcriptional activity of specific regions of DNA. The most studied of these in regeneration is the acetylation of histone lysine residues. Histones are aceytylated by histone acetyltransferases (HATs), which “opens” the chromatin to allow access to the associated genes for transcription. By contrast, histones are deacetylated by histone deacetylases (HDACs), which “closes” the chromatin and is typically repressive (Kouzarides, 2007). In this way, modulating histone acetylation can control the expression of many genes. Interestingly, peripheral axon injury, which drives regenerative gene transcription, increases acetylation of histones at the promoters of specific genes (some of which are RAGs), whereas this acetylation is not evident after central axon injury (Finelli et al., 2013; Puttagunta et al., 2014). In peripheral neurons, this is the “routine” injury response and is mediated by induced PCAF activity, a HAT protein (Puttagunta et al., 2014). This injury-induced increase in acetylation is associated with nuclear export of HDAC5, which may serve to decrease the activity of other HDAC isoforms (Cho et al., 2013). As such, augmenting histone acetylation may foster the transcription of relevant RAGs in CNS neurons. Indeed, overexpressing the HATs PCAF and p300 leads to histone acetylation at the promoters of specific genes and increased optic nerve and spinal cord axon regeneration following injury (Tedeschi et al., 2009; Puttagunta et al., 2014). Moreover, pharmacological inhibition of HDAC1 can also increase acetylation, drive RAG expression, and allows central axon regeneration (Finelli et al., 2013). Interestingly, some of the gene changes induced by these epigenetic modifiers are discordant with the standard peripheral injury response, indicating that some induced RAGs may be peripheral to the regenerative response (Finelli et al., 2013; Puttagunta et al., 2014). Both HATs and HDACs can affect the acetylation state of non-histone proteins both in the nucleus and cytosol of neurons. These modifications also play important roles in both transcriptional (i.e., p53 regulation) and non-transcriptional (i.e., microtubule dynamics) aspects of axon growth (Rivieccio et al., 2009; Tedeschi et al., 2009; Cho and Cavalli, 2012). While targeting protein acetylation with small molecules is promising, systemic administration of these drugs may be problematic as acetylation/deacetylation is important for general cellular physiology (Kouzarides, 2007). As such, there remains the need for isoform-specific modulators and more tissue/cell selective modes of drug delivery.

The reduced neuron-intrinsic injury-induced RAG response is an important reason for the failure of CNS regeneration. An interesting point to consider is why CNS neurons fail to upregulate these RAGs in response to injury. What inhibitory mechanisms prevent this response, and can we use this information to inform approaches to enhance repair? As we have discussed, the RAG program is regulated by transcription factors and epigenetic modifications. In addition to these regulatory mechanisms, post-transcriptional regulation of gene expression likely plays a critical role in regulating this program as well. The discovery of micro RNAs (miRNAs) and their role in RNA interference have added greatly to our understanding of regulation of gene expression. Based on bioinformatic predictions, miRNAs likely regulate >30% of all mammalian protein coding genes (Filipowicz et al., 2008). This mechanism appears to be important in axonal regeneration, since deletion of dicer has been shown to impair nerve regeneration in a mouse model of peripheral nerve injury (Wu et al., 2012). In addition, miRNA microarrays have identified a group of miRNAs that are expressed following injury in regenerating sciatic nerves (Strickland et al., 2011). Following spinal cord injury, more than 50 miRNAs show significant changes in expression levels. Among these is miR-145, which inhibits neurite outgrowth *in vitro* by targeting robo2 and srGAP2 (Zhang et al., 2011). In addition, both miR-133 and miR-124 are downregulated following CNS injury. Both of these are implicated in axonal regeneration, with miR-133 known to target the growth inhibitor RhoA. Dysregulation of target mRNA expression by alteration of miRNA levels may result in a failure to sustain the regenerative response, leading to failed CNS regeneration. The possibility of using miRNAs as therapeutic targets, either by anti-miRNA molecules or miRNA mimetics, offers a highly attractive ability to modulate RAG gene expression.

## Intrinsic Signals: cAMP and Conditioning Lesion Effect—What is the Role of cAMP-Mediated Transcription?

The induction of RAG expression by axon injury indicates an active process by which injured neurons sense axonal damage to activate an adaptive response. This can be achieved by the disruption of the retrograde flow of target-derived trophic signals (i.e., loss of NGF; Raivich et al., 1991; Gold et al., 1993), activation of existing or newly synthesized local (axonal) factors that are retrogradely transported (i.e., DLK, JNK, STAT3, CREB; Hanz et al., 2003; Cavalli et al., 2005; Cox et al., 2008; Ben-Yaakov et al., 2012; Shin et al., 2012), and depolarization of the axon due to the disruption of the plasma membrane (i.e., Ca^2+^ influx leading to cAMP elevation; Ghosh-Roy et al., 2010; Cho et al., 2013). Ultimately, the arrival of these signals to the cell body drives the transcriptional changes that initiate regenerative response, including the activation of transcription factors.

Interested in the molecular determinants underlying the developmental loss of regenerative capacity, the Filbin lab discovered a direct correlation between neuronal cAMP levels and the ability of neurons to regenerate. For instance, developmentally “younger” CNS neurons (i.e., early embryonic) contained high levels of cAMP and retained the ability to regenerate and overcome extrinsic axon growth inhibitors. This is in contrast to mature neurons (i.e., adult), which are incapable of regeneration and have markedly lower cAMP. In these studies, the regenerative competence of embryonic neurons *in vivo* was attenuated by pharmacological inhibition of PKA, an effector of cAMP signaling, indicating the importance of cAMP in axonal regeneration (Cai et al., 2001).

The importance of cAMP for driving axonal regeneration is not restricted to CNS neurons. Using the peripheral conditioning lesion model, the Filbin group showed that peripheral axon injury induced cAMP levels in the soma of peripheral neurons and that the *in vitro* effect of the conditioning lesion are indeed dependent on PKA (Qiu et al., 2002). Importantly, intraganglionic injection of db-cAMP, a cell membrane permeable cAMP analog, could recapitulate the conditioning lesion effect for neurons in both *in vitro* assays and *in vivo* models of dorsal column injury (Neumann et al., 2002; Qiu et al., 2002). Of note, the *in vivo* injection of db-cAMP allowed DRG neurons to overcome myelin-associated inhibitors *in vitro* in a biphasic manner. Acutely this effect was PKA-dependent (after 1 day); however, this was PKA-independent if the neurons were harvested 6 days after injection. These results indicated that the transient elevation of cAMP set forth longer-lived changes in neuronal function, which persist past the point of elevated cAMP levels. These findings were seminal as they identified a relevant, injury-induced second messenger that could initiate the regenerative process (Qiu et al., 2002).

How then does cAMP exert its benefit on axon regeneration? In mammalian cells, cAMP is generated from ATP by a family of either plasma membrane-bound or soluble adenylate cyclase (AC) enzymes. Membrane-bound ACs are regulated by the canonical heterotrimeric G-proteins, whereas soluble isoforms are activated by bicarbonate. Both soluble- and some isoforms of membrane-bound AC are also activated/regulated by Ca^2+^ (Kamenetsky et al., 2006). Axonal damage causes the depolarization of the neuron, and initiates a back-propagating Ca^2+^ transient (Ghosh-Roy et al., 2010; Cho et al., 2013); the generation of cAMP likely results from this elevation of intracellular Ca^2+^. Indeed, electrical stimulation of the peripheral nerves is sufficient to increase neuronal cAMP levels in the cell body and can mimic some of the physiological effects of the conditioning lesion (Udina et al., 2008). Recent reports suggest a prominent role for soluble AC for axon growth, as attenuating soluble AC activity, either pharmacologically or genetically, decreases PKA-dependent basal axon growth in RGCs, and prevents neurotrophin-mediated priming for axon growth on myelin-associated inhibitory substrates *in vitro*. Moreover, this inhibition of membrane-bound AC did not recapitulate these effects, indicating that soluble AC was the source of cAMP (Martinez et al., 2014; Stiles et al., 2014).

The above studies serve to link the injury event to elevated cAMP levels via a Ca^2+^-dependent regulation of AC activity. cAMP likely exerts its effects locally, affecting nearby target proteins. As such cAMP can function in both the axon and cell body compartments of neurons to regulate axon growth. In the axon/growth cone, cAMP regulates axonal guidance through the PKA-mediated inactivation of RhoA signaling and by the phosphorylation of other targets, which allows the cytoskeletal rearrangement that supports growth cone motility (Song et al., 1997; Ming et al., 2001; Kao et al., 2002; Murray et al., 2009; Cheng et al., 2011; Nicol et al., 2011; Forbes et al., 2012). Additionally, cAMP can also signal through EPAC to mediate axon guidance (Murray et al., 2009). In the cell body, cAMP may facilitate axon regeneration by inducing RAG transcription. In this way, cAMP-activated PKA can directly activate CREB-mediated gene transcription, or drive other signaling cascades that can lead to gene transcription changes (Lonze and Ginty, 2002; Gao et al., 2004; Blesch et al., 2012; Ma et al., 2014). Indeed, CREB has been linked to RAG transcription induced by the conditioning lesion, and the expression of a constitutive-active CREB protein can promote the regeneration of dorsal column sensory axons *in vivo* (Gao et al., 2004). Both cAMP and CREB are also necessary for synaptic plasticity (Kandel, 2012), highlighting that the structural plasticity of regeneration and synaptic plasticity of memory and learning utilize some of the same transcriptional substrates.

## Is cAMP Enough?

While cAMP-mediated gene transcription is an important contributor to the injury-induced RAG response, the upregulation of cAMP alone may not harness the robustness of the conditioning lesion. In studies that have directly compared axon growth following the conditioning lesion with other stimuli that increase cAMP to the same extent, conditioning lesions shows superior efficacy for axonal regeneration (Udina et al., 2008; Blesch et al., 2012). Indeed, lesion of the peripheral axon recruits more injury-induced gene transcription than increasing cAMP alone by the pharmacological inhibition of its degrading enzyme, PDE4 (Blesch et al., 2012). In light of the at least 40 transcription factors predicted to contribute to injury-induced regeneration-associated transcription, these observations suggest that cAMP-mediated transcription is a contributor to the overall response, and may control specific aspects of axon regeneration (Michaelevski et al., 2010). Accordingly, it is possible that in the absence of axonal injury, the amount of cAMP accumulated by inhibiting its degradation or by electrical stimulation is insufficient to recruit the gene transcription necessary for axonal growth comparable to the conditioning lesion. A recent study published by the Filbin group showed that overexpression of soluble AC in RGCs increased regeneration of crushed optic nerve axons. Though cAMP levels were not measured in this study, the data suggest that ongoing cAMP production, which is likely to be* supra* physiological, may increase its regeneration-promoting actions. Further, soluble AC is found in both the cell body and axon, indicating that the cAMP produced may simultaneously stimulate both compartments (Martinez et al., 2014).

RAG expression occurs on the backdrop of the activated injury-induced signaling cascades that may also have non-transcriptional effectors. As such, the expression (or overexpression) of RAGs on their own may not be optimal for promoting regeneration. For instance, accumulating data suggests that peripheral injury triggers concerted responses that *prime* the neuron to mount an effective transcriptional response. In this case, the calcium transient triggered by axotomy stimulates the epigenetic modifications necessary to facilitate transcription activated by retrograde signals (Cho et al., 2013). In addition to cAMP-PKA, other signaling molecules can act in transcription-independent manners. For instance, though JNK-mediated phosphorylation of c-Jun is necessary for its transcriptional activity, JNK isoforms also regulate axon growth through controlling cytoskeletal organization (Barnat et al., 2010; Ruff et al., 2012). Similarly, local translation initiation of β-actin, which is required for robust sensory axon regeneration, requires the Src-mediated phosphorylation of the RNA binding protein ZBP1 (Huttelmaier et al., 2005; Donnelly et al., 2011). Together, these effects likely cooperate with RAG transcription to drive axon regeneration. This makes a case for combining RAG transcription with the activation of second messengers like cAMP or Ca^2+^, in order to more closely recapitulate the injury conditioning response and drive regeneration.

Previous studies have suggested that cAMP can play a facilitative role in mediating CNS axon regeneration. In the optic nerve crush model, RGC axonal regeneration is stimulated by the release of oncomodulin by macrophages that are recruited by lens injury. cAMP is required for oncomodulin’s actions on axon growth, likely through facilitation of receptor binding (Yin et al., 2006). Given the importance of injury-induced macrophage recruitment to the DRG for injury conditioning, these factors may cooperate with cAMP to drive RAG transcription (Kwon et al., 2013; Niemi et al., 2013). Further, these manipulations seem to function in parallel to other targets for regeneration, as combining zymozan, which attracts macrophages that release oncomodulin, with cAMP and PTEN deletion synergize to allow some RGC axons to regenerate into the brain and partially restores some visual function (de Lima et al., 2012). Additionally, cAMP has been used to enhance the actions of neurotrophic factors including CNTF in the eye, and NT-3 and BDNF in the spinal cord to promote axon regeneration in systems where cAMP alone is insufficient (Cui et al., 2003; Lu et al., 2004, 2012).

## Are “Nested” Transcription Factor Networks the Key?

Though cAMP is sufficient to promote regeneration in some instances, we wanted to better understand the transcriptional determinants of cAMP’s actions. Our initial focus was CREB, as it is a well characterized transcriptional mediator of cAMP (Lonze and Ginty, 2002). Using a constitutive-active version of CREB (VP16CREB; CREB-CA) we found that either driving CREB activity in DRG neurons or application of db-cAMP alone increased axon growth on inhibitory and permissive substrates to a similar degree. Surprisingly, axon growth was significantly increased when CREB-CA-expressing neuron were treated with db-cAMP, which was paralleled by an increase in transcription of candidate RAGs. This suggested that levels of RAG transcription stimulated by CREB or cAMP alone were insufficient for the maximum observed axon growth in our paradigm. Interestingly, blocking CREB by expressing a dominant-negative variant (ACREB; CREB-DN) did not change cAMP-induced axon growth, indicating that CREB and cAMP can act synergistically, but also stimulate parallel pathways. This put forth the notion that CREB may serve as an important hub for regeneration (Table 1), but that physiological activation of CREB after injury (likely through cAMP) is insufficient to recruit the network needed to promote robust regeneration (Ma et al., 2014). This is supported by work in *C. elegans*, whose neurons exhibit robust regenerative capacity. In these neurons, cAMP upregulation elicited by axotomy-induced Ca2+ influx results in the increase of several basic leucine zipper domain transcription factors (Ghosh-Roy et al., 2010). Null mutation in the *C. elegans* homolog of CREB, crh-1, did not affect regrowth; however, it did decrease formation of ventral branches. Mutations of jun-1, however, did result in reduced regeneration. Taken together, these results suggest that CREB may not be the primary driver of cAMP-induced regeneration and that additional transcription factors may be recruited.

As CREB activity was unnecessary for cAMP’s actions, we sought the necessary transcription factor(s) downstream of CREB and cAMP that mediated axon growth. Using arginase 1 (Arg1) as a model RAG, we identified an AP1 site in the proximal promoter region. Blocking AP1 activity with a dominant-negative Fos protein (which inhibits AP1 binding to DNA) blocked both CREB-CA and cAMP-mediated axon growth; which was mirrored by Arg1 expression levels. Importantly, a constitutive-active Fos showed only modest increases in axon growth, suggesting that the AP1-controlled genes cooperate with other CREB targets to stimulate regeneration (Ma et al., 2014).

The strong induction of RAG transcription by cAMP + CREB-CA may induce* supra* physiological expression of injury-induced RAGs. Additionally, this artificial activation of CREB may recruit genes that are not physiologically induced by peripheral axon injury. For instance, CREB-CA induces high and persistent levels of c-Fos expression, which is not observed after peripheral axon injury (Herdegen et al., 1992; Haas et al., 1993). As AP1 heterodimers containing c-Fos and c-Jun have higher transcriptional activity than homodimers of c-Jun, this could further drive the transcription of AP1-dependent RAGs (Angel and Karin, 1991); however, driving AP1 activity + cAMP alone did not recapitulate the effects of CREB-CA + cAMP, reinforcing the notion that the successful axon regeneration requires a concerted and broad transcriptional response. Indeed, the strong activation of CREB recruited other previously identified hub proteins, such as ATF3, suggesting that these responses may contain functional “modules” that mediate specific aspects of axon growth (Ma et al., 2014); however, the breadth of the overall injury response may indicate redundancy at higher organizational nodes of the network. This CREB-activated, AP1-dependent gene *module* may provide significant insight to the programs necessary and sufficient to drive axon growth; its analysis by RNAseq is ongoing. As with other genome-wide techniques, many genes will be identified, though only a few will be true RAGs. Understanding the differences between these two populations may be critical for identifying the “optimal” approach for driving regeneration. It is highly probable that other *nested* transcription factor networks exist; the evaluation of these should occur in a context where changes in gene transcription can be correlated to axon growth/regeneration in order to fully elucidate their contribution to the phenotype.

## Where Do We Go From Here?

With each study of regeneration-associated transcription, we come closer to elucidating the critical programs required for axon regeneration. It is evident that the entire RAG response is difficult to recapitulate in CNS neurons due to its breadth. As such, therapeutic strategies must target more manageable critical hubs of RAG transcription or engage pathways far enough upstream to recruit the genes necessary to drive axon growth. Future strategies should combine RAG expression with non-injury induced means of increasing axon growth. This could pair genetic activation of potential upstream hubs (i.e., CREB) with the pharmacological upregulation of relevant signaling pathways (i.e., cAMP) to activate regeneration-associated transcription (Figure 1). Additionally, combinatorial strategies that increase metabolic state, recapture developmental axon growth potential, and favorably modify epigenetic state of RAGs could function in synergy with RAG expression to further promote axon growth following injury. With the proper experimental design and the explosion of “big-data” analysis of transcriptomics data, we may now be poised identify the “regeneration-associated hubs” to target, and make good on the promise of Filbin’s seminal observations on cAMP. We may find that it isn’t necessary to completely recapitulate the peripheral-injury response in order to effect significant regeneration.

## Conflict of Interest Statement

The authors declare that the research was conducted in the absence of any commercial or financial relationships that could be construed as a potential conflict of interest.
